# Upper limbs dysmetria caused by cervical spinal cord injury: a case report

**DOI:** 10.1186/1471-2377-9-50

**Published:** 2009-09-24

**Authors:** Hsun-Chang Lin, Chun-Hung Chen, Gim-Thean Khor, Poyin Huang

**Affiliations:** 1 Department of Neurology, Kaohsiung Medical University Hospital, Kaohsiung Medical University, Kaohsiung, Taiwan, Republic of China

## Abstract

**Background:**

Upper limbs dysmetria caused by spinal cord injury is very rare. We will discuss the associated mechanism in our articles.

**Case presentation:**

A 51-year-old male had sudden onset of weakness, dysmetria over bilateral upper limbs and ataxia after he fell accidentally. Brain magnetic resonance imaging (MRI) revealed no specific findings. C-spine MRI revealed C1 myelopathy and C4-6 spinal cord compression by bulged disc. The symptoms subsided after surgical intervention.

**Conclusion:**

Sudden onset of upper limbs dysmetria is a sign of dysfunction in cerebellum and its associated pathway. However, lesion in spinal cord can also cause cerebellar signs such as dysmetria.

## Background

Upper limbs dysmetria is thought to be a cerebellar sign, and is also seen in some metabolic diseases, heavy metal poisoning or drug effect. Sudden onset of dysmetria is considered to be the dysfunction of cerebellum and its associated pathway. [1-6] However, the mechanism and the conducted pathway are not completely understood. Herein, we report a case of sudden onset dysmetria secondary to cervical cord injury and discuss the possible mechanism.

## Case presentation

A 51-year-old male had no systemic disease or medication history before. Dysarthria was noted since his childhoods. There was no functional impairment in his daily activity including walking, writing, using chopsticks or combing his hair.

He fell down when he was dancing and bumped on floor with his buttock. Weakness of both upper limbs and neck pain were noted without loss of consciousness. He was brought to our emergent department immediately. Neurological examination revealed intact cranial nerve function. Weakness over bilateral upper limbs was found. (Muscle power of right proximal/distal: 4/3, Left proximal/distal: 4/4 in Medical Research Council grading) There was no weakness over lower limbs. Sensory test was intact either in pin-prick or proprioception. However, bilateral dysmetria was noted in finger-nose-finger test, especially the left hand, even when his elbows were held. The dysmetria was confirmed by three neurologists, and was out of proportion to his weakness. In the Heel-knee-shin test, there was no dysmetria. Ataxia was seen while he was standing even the muscle power was full in his legs. The deep tendon reflex of four limbs was increased. When checking Babinski signs, bilateral dorsiflexion was noted. The presence of neck pain and bilateral upper limbs weakness may be resulted from a cervical spine lesion. Bilateral upper limbs dysmetria was not so common clinically. Considering the possibility of a vascular episode in the cerebellum, which resulted in his falling, a brain image was also arranged even when there were no other clinical signs of brain lesion at time of examination. C-spine X-ray was done and mild degenerative disc disease at C4-5 was noted, and there was no definite lesion seen in brain Computed tomography (CT).

After admission, Methylprednisolone therapy was initiated about 5 hours after the trauma. The dose was 100 mg/hour (1.6 mg/kg/hour) for 48 hours without any additional bolus dose.

**Figure 1:**
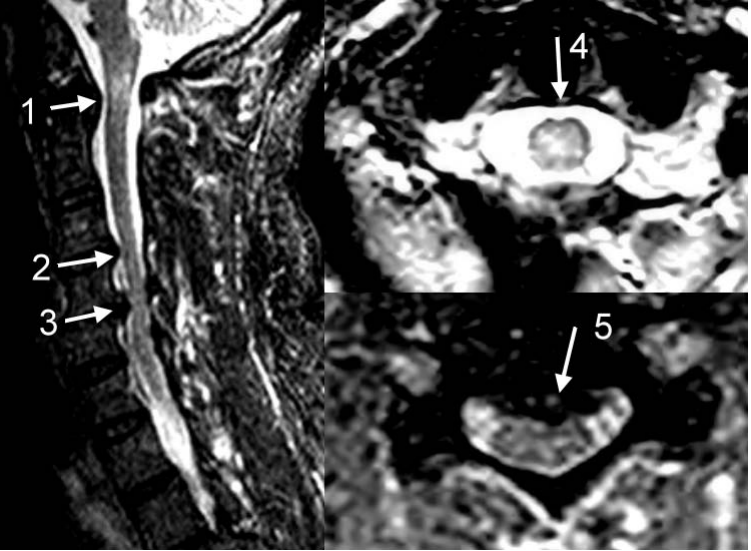
**T2 image of Cervical spine MRI**. Arrow 1: Hyper dense lesion at C1 level. Arrow 2 and 3: Bulged disc over C4-5, C5-6 with spinal cord compression. Arrow 4: Cross section of C1 level. The hyper dense lesion distributes over the central area and bilateral lateral column. Arrow 5: Bulged disc of the C5-6 with cord compression in the anterior aspect

We arranged brain MRI, but there was only old ischemic-gliotic change over left frontal lobe without any gross lesion over brainstem or cerebellum. The C-spine MRI was also arranged. There was hyper intense lesion over C1 spinal cord, and bulged discs over C4-5, C5-6 with spinal cord compression were seen. (Figure 1) The hyper intense lesion at C1 was distributed over the central area and bilateral lateral column. The cord compression at C4-6 was in the anterior aspect. C1 myelopathy and C4-6 spinal cord compression by bulged discs were impressed. And the hyper intense lesion at C1 was supposed to be caused by the cord injury. After the steroids treatment, there was partial improvement but certain portion of the weakness and dysmetria persisted. We then consulted neurosurgeon for surgical intervention and an operation was performed nine days after the trauma. After the operation, his weakness and dysmetria further improved and the patient could walk without obvious ataxia. Written consent was obtained from the patient for publication of study.

## Conclusion

It was difficult to localize for the sudden onset of weakness, dysmetria over both upper limbs and ataxia. Cerebellum, superior cerebellar peduncles, red nucleus, and thalamus are involved in the associated conduction and mechanism of dysmetria reported in previous studies. [1-6] But in this case, there is no definite finding over the cerebellum, brainstem, or thalamus in brain MRI. C1 myelopathy and C4-6 cord compression were seen in cervical spine MRI.

We analyze the pathway associated to the cerebellum in the spinal cord. We can divide them into input and output pathway. In the input pathway from spinal cord, the spinocerebellar tract conveys the proprioception from extremities. It hence influences the coordination and the balance. In the somatotopic representation of the spinal cord, fibres coming from the upper legs are conveyed laterally. Fasciculus gracilis and cuneatus are also responsible for limbs position. From the clinical point of view, ataxia may be present with dysfunction of these spinal cord tracts. Actually, the patient had ataxia in the standing position but sensory disturbances were not evident, although they are usually presented with cord ataxia. In previous studies, [7,8] there were nine patients with malignant disease with thoracic spine metastasis. The epidural spinal cord compression was noted. Ataxia and lower limbs dysmetria without weakness were supposed to be the spinocerebellar tract dysfunction in the articles.

In the cerebellar output pathway, we can divide into three parts as the lateral hemisphere, the intermediate hemisphere, and vermis plus flocculonodular lobe. The interposed neclei in the intermediate hemisphere projects via the superior cerebellar pudencle to the contralateral thalamus and then projects to the motor and associated cortex, and then influences the corticospinal tract. The interposed neclei also projects to the contralateral red nucleus and then influence the rubrospinal tract. There is a double cross relation between the cerebellum and spinal cord. The rubrospinal tract starts from the red nucleus and crosses via the ventral tegmental decussation. And it runs through the medulla and terminates at the level of cervical cord.

The role of rubrospinal tract was not clear in human beings. But the cerebellar output pathway, especially from the intermediate part, influences it. The intermediate hemisphere is involved in ongoing movement of distal extremities. So the rubrospinal tract may play a role in the coordination of limbs. Besides, the rubrospinal tract runs closely to the corticospinal tract in the lateral motor systems of the spinal cord. A lesion in cervical cord may cause both corticospinal and rubrospinal tract deficits. This may explain why his weakness and dysmetria occurred together in this case. We had reviewed another study, [9] they fulgurated the superior cerebellar peduncles in two monkeys. Cerebellar ataxia and ataxic tremor were noted. Scratching movements were also dysmetric. They did fulguration of rubrospinal tract at the medulla level in the same two monkeys after three week. The tremor was more severe when they held something. Cerebellar ataxia and ataxic tremor in movement were unchanged. This may be the evidence that the rubrospinal tract plays a role in the coordination of extremities. However, more proof may be needed in human beings.

In the patient's Cervical MR, there were C1 hyperdensity and C4-6 cord compression noted. MR hyperdensities in spinal cord may locate within or far from the site of focal spondilotic alternations. Previous report had reported that there was girdle sensation located from T3 to T11 level in the patients with cervical compressive myelopathy. [10] It was explained by due to ischemia in the watershed zone of spinal cord. Compression of anterior spinal artery at the cervical level might result in ischemic change of watershed zone in spinal cord, even in other spinal levels.

The patient improved after surgical intervention. There were two explanations for his improvement. First, the improvement of dysmetria and weakness could be a coincident effect of steroids. The effect of methylprednisolone on motor function could be observed from days to months after medication. [11] Second, the clinical feature of early cord compression is dominant by motor function impairment. It may be resulted from the compression of vessels supplying the spinal cord, causing edema and poor capillary circulation in the watershed zone which includes the corticospinal pathway. [12] In this patient, the compression of C4-C5/C5-C6 from the anterior aspect may caused ischemia over the watershed zone of C4-6 level, which involves the corticospinal and rubrospinal tracts. For this reason, we consulted neurosurgeon for surgical treatment of C4-C5/C5-C6 spine decompression. His clinical symptoms also got improved after the surgical intervention. This may be resulted from relief of spinal artery compression. The surgery also relieved the ischemia change in the watershed zone at the level of focal spondilotic alternations and even at other levels. However, whether the C1 hyperdensity is related to the watershed zone ischemia from C4-C6 compression can not be confirmed. A post-operative cervical MR could probably give some insights on the role of C1 lesion and C4-C5/C5-C6 cord compression.

In clinical practice, it need deepen and detailed clinical observation and evaluation to clearly distinguish a true dysmetria co-existing with weakness and ataxia. Sudden onset of dysmetria is usually a sign of dysfunction in cerebellum and its associated pathway. But in this case report, we can conclude that dysmetria could be a sign of spinal cord lesions. The presence of dysmetria associated with weakness and ataxia can lead to the hypothesis of corticospinal, spinocerebellar, rubrospinal and posterior columns involvement. We tried to explain the important role of watershed zone ischemia and rubrospinal tract involvement in this case's clinical symptoms. More cases are needed to support our hypothesis.

## Competing interests

The authors declare that they have no competing interests.

## Authors' contributions

CHC and GTK reviewed the references and proposed the possible mechanism in this article. HCL and PH participated in the discussion and interpretation in the associated mechanism in this article. HCL and PH are involved in drafting and revising the manuscript. All authors read and approved the final version of the manuscript.

## Pre-publication history

The pre-publication history for this paper can be accessed here:


